# Structured reports of pelvic magnetic resonance imaging in primary endometrial cancer: Potential benefits for clinical decision-making

**DOI:** 10.1371/journal.pone.0213928

**Published:** 2019-03-25

**Authors:** Yi Liu, Zonghao Feng, Shengtang Qin, Jiejin Yang, Chao Han, Xiaoying Wang

**Affiliations:** 1 Department of Radiology, Peking University First Hospital, Beijing, China; 2 Department of Obstetrics and Gynecology, Peking University First Hospital, Beijing, China; University of Wisconsin-Madison, UNITED STATES

## Abstract

**Background:**

Although evidence is increasing that the implementation of structured reports (SRs) may increase the standardization of reports and improve communication between radiologists and end-users, it is unclear whether these alternative formats of Chinese radiological narratives are appealing or even acceptable to radiologists and clinicians.

**Objective:**

To compare the effect of SRs and non-structured reports (NSRs) of pelvic magnetic resonance imaging (MRI) in patients with primary endometrial cancer on referring gynecologists’ satisfaction, further decision-making and efficiency.

**Methods:**

Forty-one patients with histologically proven endometrial cancer were included in this study. SRs and NSRs for local MRI staging of endometrial cancer were generated for all subjects. NSRs were generated during clinical routine practice. The same 41 uterine studies were reviewed by the same radiologist using structured reporting system after a period of time. Two radiologists compared SRs on the number of key features related to cancer staging and writing efficiency with NSRs together. Five gynecologists filled in questionnaires regarding satisfaction with content, clinical usefulness, report’ quality and time consumption. Statistical analysis included Kendall’s W test, paired-sample *t* test and Wilcoxon signed rank test.

**Results:**

There was no significant difference in the number of key features in NSRs comparison to SRs (*p* = 0.055). A statistically significant difference was observed in the satisfaction with linguistic quality for NSRs versus SRs by three gynaecologists (reader 1: 4.02 vs. 4.63, *p* = 0.002; reader 3: 3.86 vs. 4.02, *p* = 0.035; reader 4: 4.05 vs. 4.27, *p* = 0.024). The radiologist spent less time finishing SRs compared with NSRs (727.22 ± 38.42 sec vs. 616.44 ± 60.00 sec, *p* = 0.037).

**Conclusions:**

The application of SRs significantly increased the value of female pelvic MRI reports by increasing radiologists’ work efficiency and gynaecologists' satisfaction.

## Introduction

Endometrial cancer is a common malignancy with high mortality and morbidity rates being the sixth most common cause of cancer-related deaths in females worldwide [1]. The presence of abnormal uterine bleeding is suggestive of a diagnosis of endometrial cancer after ruling out cervical disease by gynecological examination. The initial staging of endometrial cancer is essential for further clinical treatment decisions and thus for the prognosis of patients with endometrial cancer. Surgery is the criterion standard treatment for endometrial cancer if the patients’ conditions and stage of disease permit it [2,3].

For staging of endometrial cancer, dynamic contrast-enhanced magnetic resonance imaging (MRI) is the main tool for diagnosis because of its excellent soft tissue contrast and multiplanar capability [4]. MRI allows for an accurate assessment of tumor size, localization, infiltration into surrounding structures, and the depiction of locoregional lymphadenopathy, helping for a correct surgical planning and risk stratification of patients who would potentially benefit from preoperative irradiation or systemic chemotherapy. Overstaging would lead to unnecessary radiotherapy or chemotherapy and understaging may increase the risk of tumor recurrence [3]. Therefore, a correct and unambiguous radiological staging is crucial for further clinical planning and the prognosis of patients with histologically endometrial cancer.

This ultimate goal of radiology report is to be understood easily and help guide patient care. Despite the importance of radiology reports, they have historically been created by using non-structured text; that is, the radiologist dictates in narrative style as he or she deems appropriate [5]. As the result, the content of radiology reports is non-standardized and some key features are omitted sometimes by radiologists due to a lack of knowledge on the exact details the referring clinicians are expecting, and so it may not serve the needs of patients and referring physicians so well [6,7]. This is why the necessity to create structured reports (SRs) has been widely discussed in recent years. Several recent studies revealed that SRs were much easier to understand and served better the needs of patients and referring physicians [8–10]. A reduction in omissions of findings was detected. Furthermore, SRs allow gathering of organized data about patients and conditions, which contributes to our ability to mine these data effectively [11].

Although evidence is increasing that the implementation of SRs may increase the standardization of reports and improve communication between radiologists and end-users, it is unclear whether these alternative formats of Chinese radiological narratives are appealing or even acceptable to radiologists and gynaecologists. The goal of this study was to determine (1) the integrity of key information, (2) the convenience of extracting key information related to staging, (3) the gynecologists’ perception of clinical usefulness, (4) the satisfaction level of gynecologists in linguistic quality and overall quality and (5) the efficiency of writing reports and reading reports of SRs compared with NSRs based on Chinese text.

## Materials and methods

### Patient population

This study was a retrospective study. The study protocol was approved by the responsible institutional review board of the Peking University First Hospital with waiver of informed consent. 41 uterine MRI reports of patients with histologically proved primary endometrial cancer before implementation of the structured reporting system (June 15, 2017) were continuously selected from our radiology information system (RIS) and picture archiving and communication system (PACS). All these patients met the following inclusion criteria: histologically proved primary endometrial cancer; without previous treatment of uterus and bilateral attachment (such as, surgery, external irradiation or chemotherapy) before MRI scanning; the objective was to determine the grade of endometrial cancer; images were available for evaluation. Authors had access to information that could identify individual participants during and after data collection.

### MRI protocol

MRI examination was performed on a 3T scanner (GE Discovery 750, GE Healthcare, Milwaukee, WI, USA) using a dedicated MRI endometrial cancer staging protocol, which including sagittal, axial and coronal T2-weighted fast spin-echo, axial T1-weighted fast spin-echo, axial diffusion-weighted imaging echo planar imaging and sagittal dynamic contrast-enhanced T1-weighted fast spin-echo.

### Study design and endometrial MRI reports selection

**Fig 1:**
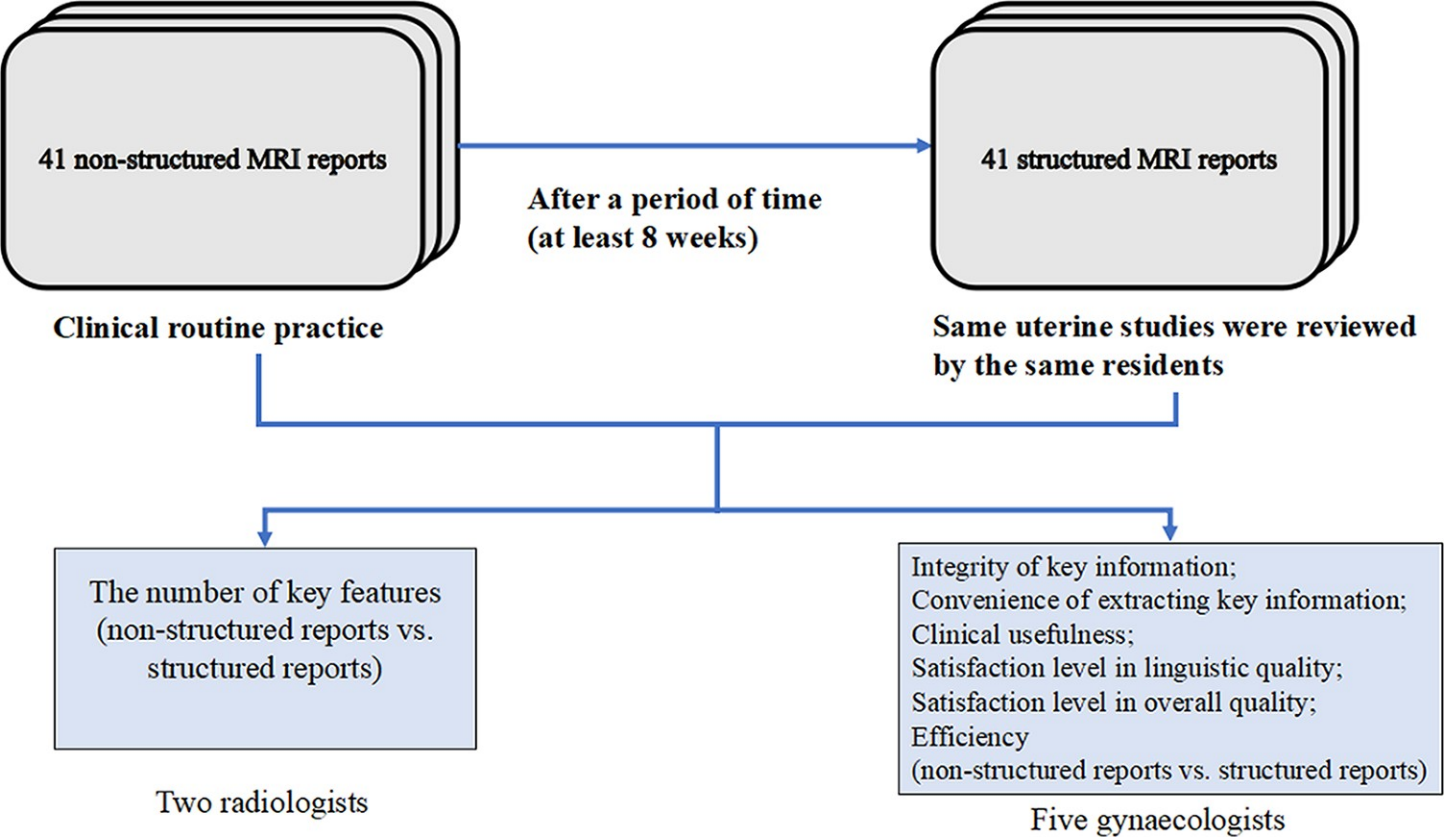
The overview of our study. 41 NSRs were generated under clinical routine practice. The same 41 uterine studies were reviewed by the same radiologist after a period of time. The reports were provided to 2 radiologists and 5 gynecologists for further evaluation.

Two different types of radiological reporting were evaluated: NSRs and SRs of pelvic MRI for all 41 MRI studies. All NSRs were generated under clinical routine practice by 11 radiologists including a 2nd-year resident, four 3rd-year residents, four 4th-year residents and two 5th-year residents and retrospectively taken from our RIS. After the NSRs were written and submitted by residents, the abdominal imaging professional radiologist would review the report, then the reports would be issued. 41 uterine studies were reviewed by the same radiologist that performed the initial interpretation, generating SRs after a period of time. No access to the NSRs was possible when generating the same cases using the SR template. Similarly, after the SRs were written and submitted by residents, the abdominal imaging professional radiologist would review the report. The SRs were not being issued as they were only for the purpose of this study. A break of at least 8 weeks was completed after completion of NSRs to avoid recall bias for writing of SRs. The reports were provided to 2 experienced radiologists with 8-years and 12-years experience in female pelvic imaging, respectively, and 5 gynecologists (reader 1 was a staff clinician with 9 years of experience, reader 2 was a staff clinician with 11 years of experience, reader 3 was a staff clinician with 12 years of experience, reader 4 was a staff clinician with 10 years of experience, reader 5 was a staff clinician with 8 years of experience) for further evaluation. The radiologists and gynecologists involved in reviewing the reports were different to those involved in the development of the SR template. The overview of the study is shown in Fig 1.

### SRs MRI template

The MRI template report for endometrial cancer was developed in consensus by two gynecologists specialized in treating patients with endometrial cancer and two radiologists with 5 years and 22 years of experience who commonly interprets MRI examinations in patients with endometrial cancer. The template included all information deemed necessary for staging of endometrial cancer according to FIGO 2009 [12,13] and was implemented in the RIS on June 15, 2017. The template is separated into clinical evaluation, technical evaluation, findings and impression; furthermore, the findings section is subdivided into overall assessment, lesions assessment and auxiliary findings. Various standardized entries, with associated default checked results that describe normal or the most common findings, were included in SR template. The diagnostic impression is automatically generated based on the description of findings. However, it also possible to adjust text phrases and add additional sentences manually by offered text boxes at any section of the template.

The structured report templates were entered into RIS in normal workflow. Before the initial generation of SRs, all radiologist-in-training and radiologists were trained on how to use the SRs template under a radiologist’ supervision.

### Evaluation of key features and efficiency by radiologists

The 41 SRs and 41 NSRs were reviewed by two radiologists with 8-years and 12-years experience in pelvic MRI. They evaluated and compared SRs and NSRs for key features deemed important for staging of endometrial cancer and were not involved in reading the MRI studies. Each feature was considered present if it was mentioned in the report (regardless of whether the finding was positive or negative) and absent if it was not mentioned.

All 18 key features were listed and assessed by the radiologists. For overall assessment, key features were position and size of uterine, if mass is visible in uterine cavity or not and the size or largest diameter of mass. For lesion assessment, key features were myometrial invasion by tumor, the invasion of cervical stroma, surrounding structure, serosa of the corpus uteri, adnexa, vagina, parametrial tissue, pelvic lymph node, para-aortic lymph node, bladder, bowel mucosa, distant metastasis, intra-abdominal lymph nodes and inguinal lymph nodes.

The time radiologists spent generating each report was automatically recorded by RIS or the SR system.

### Evaluation of reports by gynecologists

Retrospectively deidentified MRI reports were provided to gynecologists. Five gynecologists independently evaluated the reports blinded to all clinical data and other relevant reports. After 8 weeks, to avoid recall bias for the assessment of NSRs, gynecologists evaluated SRs. The time from initial read to final read of radiology report by the gynecologist was recorded and compared. The time of distractions, concern for the patient’s clinical outcome and review of the clinical records was not recorded for reading NSRs or SRs. For each evaluation, reader filled in the general questionnaire (S1 Appendix) including the following questions: (1) whether the key questions of the referring physician have been answered? (1, yes; 2, results are ambiguous and needing further consultation with radiologists; 3, no) (2) whether the information extraction was convenient? (1, yes; 2, uncertain or neutral; 3, no) (3) if they had enough information to make an adequate clinical decision? (1, yes; 2, further consultation with radiologists; 3, no) (4) how satisfied are you with the linguistic quality of this report? (on a Likert scale ranging from 1 (dissatisfied) to 5 (very satisfied) (5) how satisfied are you with the overall quality of this report? (on a scale from 1 to 5) (6) how much time was needed to read and understand the reports?

### Statistical analysis

All statistical analysis was performed using SPSS version 20.0 (IBM Corp., Armonk, NY, USA). The normal distribution was assessed using the Kolmogorov-Smirnov test. Variables are reported as mean ± standard deviation for variables with normal distribution and as median and 25/75 percentile for variables with non-normal distribution. Kendall’s W test was used to assess agreement between observers. Good inter-observer agreement was noted between the readers when Kendall’s W ≥ 0.50. Paired-sample *t* test was used to compare the average number of key features present in NSRs and SRs. The Wilcoxon signed rank test was carried out to determine differences in content, information extraction, clinical usefulness, linguistic and overall quality between NSRs and SRs. Paired-sample *t* test was used to compare the time consumption of radiologists to finish NSRs and readers to read and understand NSRs with the time consumption to SRs. *P* value < 0.05 was considered statistically significant.

## Results

### Population

A total of 41 cases of histologically proved primary endometrial cancer were identified, of which 9 (21.95%) were diagnosed as stage IA, 17 (41.46%) as stage IB, 11 (26.83%) as stage II, 3 (7.32%) as stage IIIC1 and 1 (2.44%) as stage IIIC2 according to FIGO 2009. The mean age of the patients at the time of diagnosis was 55.8 ± 13.3 years, age range 27 to 91 years.

### Evaluation of key features

For all of the 41 pelvic MRI studies, the mean number of key features was 13.78 ± 0.72 (mean ± SD; range, 12–15) in NSRs and 14.20 ± 1.03 (mean ± SD; range, 11–15) in SRs. There was no significant difference in the number of key features in NSRs comparison to SRs (*p* = 0.055).

### Evaluation of NSRs and SRs by gynecologists

All of the 410 questionnaires (41 for NSRs and 41 for SRs sent to each gynecologist) were well received (100% reply rate).

**Fig 2:**
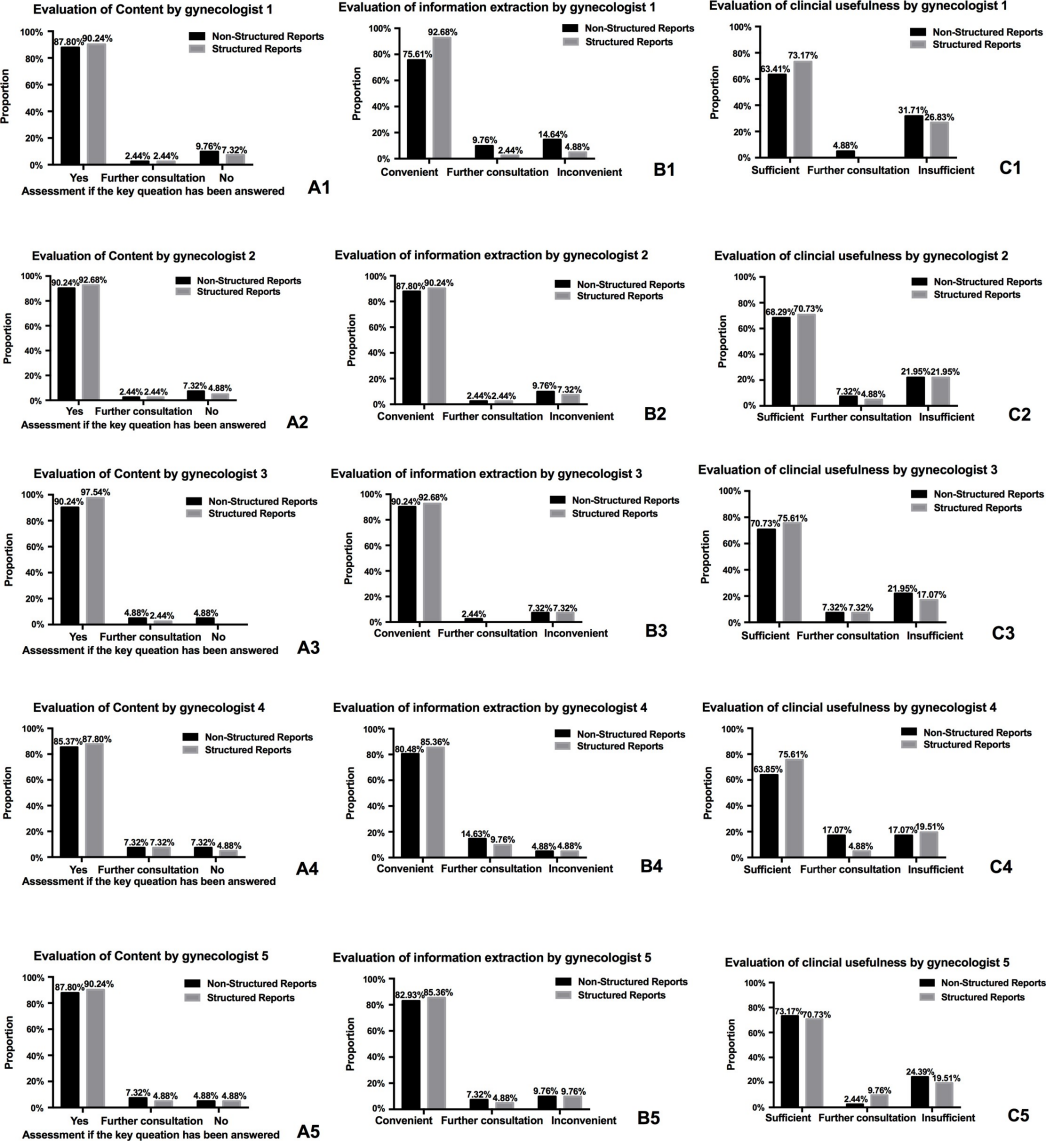
Bar graphs of reports evaluation raging from gynecologists. (A1-A5) Bar graphs of distribution of content evaluation raging from three gynecologists. (B1-B5) Bar graphs of distribution of evaluation of information extraction raging from three gynecologists. (C1-C5) Bar graphs of distribution of clinical usefulness raging from three gynecologists.

Whilst satisfaction with content was higher for SRs compared to NSRs, this difference did not reach statistical significance (reader 1: 1.22 vs. 1.17, *p* = 0.708; reader 2: 1.17 vs. 1.12, *p* = 0.157; reader 3: 1.14 vs. 1.02, *p* = 0.059; reader 4: 1.22 vs. 1.17, *p* = 0.480; reader 5: 1.17 vs. 1.15, *p* = 0.862) (Table 1). All gynecologists found a greater percentage of key questions were answered by the SRs versus the NSRs, as demonstrated in Fig 2A.

**Table 1:** Differences between NSRs and SRs evaluated by gynecologists.

Topic of evaluation question	Gynecologist 1				Gynecologist 2				Gynecologist 3				Gynecologist 4				Gynecologist 5			
	NSR*	SRs**	*Z* value	*P* value	NSRs*	SRs**	*Z* value	*P* value	NSRs*	SRs**	*Z* value	*P* value	NSRs*	SRs**	*Z* value	*P* value	NSRs*	SRs **	*Z* value	*P* value
Answered key questions	1.22	1.17	-0.375	0.708	1.17	1.12	-1.414	0.157	1.14	1.02	-1.890	0.059	1.22	1.17	-0.707	0.480	1.17	1.15	-0.173	0.862
Convenience of information extraction	1.39	1.12	-1.675	0.094	1.22	1.17	-0.375	0.708	1.17	1.14	-1.000	0.317	1.24	1.20	-1.000	0.317	1.27	1.24	-0.173	0.862
Clinical usefulness	1.68	1.54	-0.571	0.568	1.56	1.54	-1.000	0.317	1.50	1.41	-0.527	0.598	1.51	1.44	-0.351	0.726	1.51	1.49	-0.228	0.820
Linguistic quality	4.02	4.63	-3.047	0.002	3.90	3.98	-1.732	0.083	3.86	4.02	-2.111	0.035	3.95	4.32	-2.266	0.023	4.07	4.17	-0.779	0.436
Overall quality	3.95	4.34	-1.706	0.088	4.05	4.07	-0.302	0.763	4.05	4.07	-0.209	0.835	3.98	4.05	-0.660	0.509	3.98	4.02	-0.443	0.658

*NSRs: non-structured reports

**SRs: structured reports.

No statistically significant difference was seen for the convenience of information extraction for NSRs versus SRs (reader 1: 1.39 vs. 1.12, *p* = 0.094; reader 2: 1.22 vs. 1.17, *p* = 0.708; reader 3: 1.17 vs. 1.14, *p* = 0.317; reader 4: 1.24 vs. 1.20, *p* = 0.317; reader 5: 1.27 vs. 1.24, *p* = 0.862) (Table 1). Whilst the information extraction from SRs were considered to be more convenient compared to NSRs by reader one, this difference did not reach statistical significance. All gynecologists found the majority of NSRs and SRs were convenient for information extraction, as demonstrated in Fig 2B.

Concerning clinical usefulness, there was no statistically significant difference between NSRs and SRs for all readers (reader 1: 1.68 vs. 1.54, *p* = 0. 568; reader 2: 1.56 vs. 1.54, *p* = 0.317; reader 3: 1.50 vs. 1.41, *p* = 0.598; reader 4: 1.51 vs. 1.44, *p* = 0.726; reader 5: 1.51 vs. 1.49, *p* = 0.820) (Table 1). For reader 1, 31.71% of NSRs were considered to be insufficient for decision making, whilst 4.88% required further consultation with the radiologist; 26.83% of SRs were considered to be insufficient. The other readers had similar evaluations. (Fig 2C).

**Fig 3:**
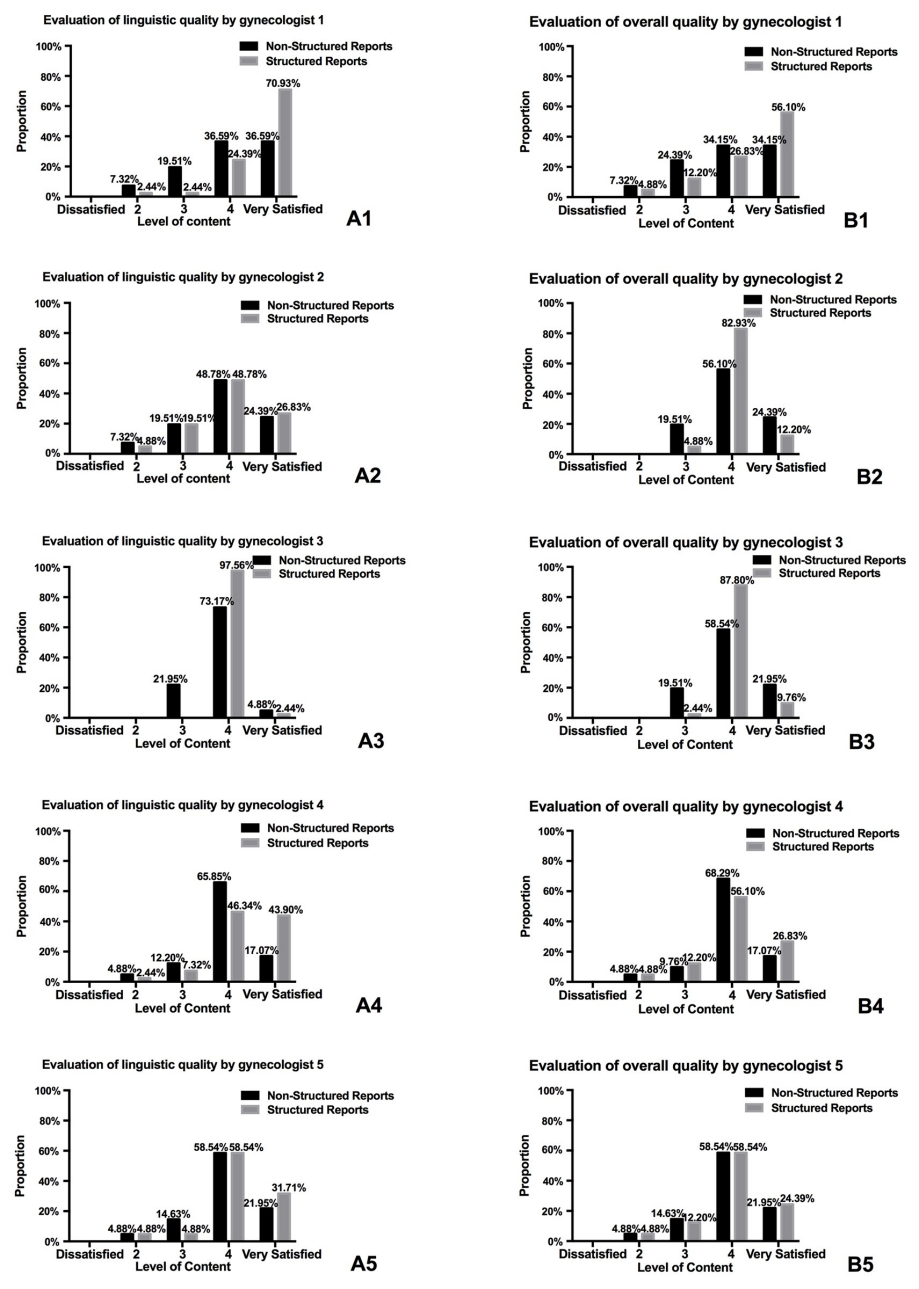
Bar graphs of distribution of satisfaction of linguistic quality and overall quality raging from gynecologists. (A1-A5) Bar graphs of distribution of satisfaction of linguistic quality raging from three gynecologists. (B1-B5) Bar graphs of distribution of satisfaction of overall quality raging from three gynecologists.

When reports were evaluated, a statistically significant difference was observed in the satisfaction with linguistic quality for NSRs versus SRs by three gynaecologists (reader 1: 4.02 vs. 4.63, *p* = 0.002; reader 3: 3.86 vs. 4.02, *p* = 0.035; reader 4: 3.95 vs. 4.32, *p* = 0.023) (Table 1). Fig 3A shows the distribution of ratings for satisfaction with linguistic quality for NSRs and SRs and all gynecologists found a greater percentage of the high end of scale (4–5 range) of SRs compared to NSRs.

Concerning overall quality of reports, no statistically significant difference was shown between NSRs and SRs for all readers (reader 1: 3.95 vs. 4.34, *p* = 0.088; reader 2: 4.05 vs. 4.07, *p* = 0.835; reader 3: 4.05 vs. 4.07, *p* = 0.835; reader 4: 3.98 vs. 4.05, *p* = 0.509; reader 5: 3.98 vs. 4.02, *p* = 0.658) (Table 1). Readers’ ratings clustered around the high end of scale (4–5 range) both for NSRs and SRs (Fig 3B).

In regarding to content, information extraction, clinical usefulness, linguistic quality, overall quality of NSRs and information extraction and clinical usefulness of SRs, the inter-observer reliability showed almost perfect agreement, with a Kendall’s W value of 0.921, 0.837, 0.944, 0.856, 0.888, 0.846 and 0.957. In regarding to content, linguistic quality, overall quality of SRs, there was substantial agreement, with a Kendall’s W value of 0.792, 0,700 and 0.734.

### Evaluation of efficiency

A statistically significant difference was observed in the time of finish for NSRs versus SRs by the radiologist (727.22 ± 38.42 sec vs. 616.44 ± 60.00 sec, *p* = 0.037), whereas no significant difference was observed in terms of time to read and understand NSRs versus SRs by gynecologists (reader 1: 34.93 ± 1.08 sec vs. 34.17 ± 1.08 sec, *p* = 0.615; reader 2: 35.12 ± 1.32 sec vs. 33.56 ± 1.13 sec, *p* = 0.238; reader 3: 34.39 ± 1.24 sec vs. 31.17 ± 1.01 sec, *p* = 0.063).

## Discussion

Endometrial cancer is generally staged according to the International Federation of Gynecology and Obstetrics (FIGO) system [12,13]. It is based on total hysterectomy and bilateral salpingo-oophorectomy. Accurate local staging of endometrial cancer using MRI is of high importance because it is essential for determining the correct treatment approach. MRI using high spatial resolution T2-weighted and contrast-enhanced T1-weighted images offers tumor delineation for staging of endometrial cancer with high accuracy and assists in clinical decision-making.

Over the last decade, the complexity of oncological radiology reporting has increased significantly due to the fast-growing amount of diagnostic imaging parameters that are included in oncologic guidelines and exact radiological assessment and recording of these key features is needed to correctly guide surgeons in their clinical decision making. Despite the important of the oncological report, it has historically been created by free-text style that may lead to inter-observer variability in the content of reports. One way to substantially reduce the variability is by the use of SRs with standardized format and lexicon rather than NSRs. This standardization may serve to increase report completeness and effectiveness. The standardized reporting system are preferred by radiologists and gynaecologists because it can potentially improve completeness, consistency and quality of radiology reports and thus influencing the clinical decision [10,14,15].

Our study demonstrated that there was no statistically significant difference in the number of key features in NSRs compared to SRs, but the difference between them was borderline significant. To be more specific, the number of key features in SRs was larger than that in NSRs. The major task faced by gynecologists having made a diagnosis of cancer is to determine the most effective therapy and formulate the prognosis of patients. Dedicated factors such as the extent of invasion, and distant metastases directly impact on the option of treatment method; surgical decisions require precise localization of the primary tumor and metastases. Given the complexity of such clinical decisions, the implementation of key features for clinical planning into SRs is of utmost important. The key features associated with staging are embedded in the template of SRs. The use of SRs alleviates the need to remember every key feature in a report for a given disease process. We demonstrated that important key features for clinical planning, such as depth of myometrial invasion and para-aortic, intra-abdominal and inguinal lymph nodes, were more frequently reported in SRs than in NSRs. Those key features are necessary in staging endometrial cancer and closely related to clinical planning, for example, systemically therapeutical approach with palliative surgery is suitable for stage IV (having intra-abdominal metastases and/or inguinal lymph nodes [16].

There was no significant difference in satisfaction with content between NSRs and SRs, but one gynaecologist found that the difference between them was borderline significant. In detail, the satisfaction of content in SRs was higher than that in NSRs. The SRs template contained various standardized entries which made the content of SRs more complete and clearer. Gynecologists also found that the linguistic quality of SRs were better in comparison to NSRs. The SRs were created by using a new software tool that translates clickable and optional decisions into predefined text phrases that were interdisciplinary generated by the experienced radiologists and gynecologists. The predefined text phrases were stored in the operation interface of the SRs. When the radiologists wrote reports, they only need to select these words with the mouse, and then those words can directly be added to the reports. In addition, our research was based on Chinese reports. Chinese text is characterized by complexity and diversity [17]. In English, the meaning of the word is clear and the possibility of confusion is small due to the difference in the recording format. However, the Chinese character is written in different meanings even though the same word is used. The structure of the SRs template was standardized and various standardized entries were included in the SRs template which significantly reduce the possibility of confusion.

Other studies have shown that clinicians seem to prefer such structured reporting because this approach uses medically correct phrases and may reduce variability and error rate, such as misspelling or grammatical mistakes [18,19].

Although some radiologists feel that SRs are overly constraining and more time consuming to complete [20], our study demonstrated that the structured approach was less time-consuming for radiologists in comparison to NSRs. An important reason for improvement of finishing reports in this study might be the use of clickable and optional mode, which makes it easier for radiologists to finish SRs in a time economic manner. Furthermore, the cancer staging is automatically generated based on the description of findings, so radiologists need not spend time judging the cancer staging. For viewing time, our study showed that there was no significant difference between NSRs and SRs. This finding was similar with the results of prior studies [21,22], in which researchers found the report format did not influence the report viewing time, despite nearly uniform clinician preference for the structured format.

Our study found radiologists spent less time generating SRs compared with generating NSRs. The report generation process in China is different from that in Western countries and the United States. Chinese radiologists are used to generating reports by typing on the keyboard rather than using a dictation system in their daily work. Checking a box on a form was quicker than typing the findings. The results mean that the using of SRs had an increase on work efficiency. However, it is not at all clear that this finding would be obtained in situations where dictation and speech recognition (or transcriptionists) are used. Dictation can be done quickly without taking eyes off the image; looking for the proper box to type might take more time and be distracting. We would carry out the relevant research after our department introduces a dictation system. Our study serves as a precedent for the exploration of SRs based on Chinese narratives and similar researches have just started.

There were several limitations to this study. First, only 5 gynecologists evaluated the reports. However, the goal of this study was to assess the gynecologists’ satisfaction of SRs in comparison to NSRs, not the ability of gynecologists to make clinical decisions. In addition, the potential effect of complete and unambiguous SRs in patients with primary endometrial cancer on the choice of clinical decision requires further investigation in the long term. The follow-up of patients should be considered in future studies in order to investigate the impact of report type on patient outcomes.

## Conclusion

In conclusion, the application of SRs increased the value of MRI reports in primary endometrial cancer by improving gynecologists’ satisfaction, and decreasing report writing time. More extensive, multicenter studies are needed on the effect of SRs on users’ satisfaction and clinical decision-making.

## Supporting information

S1 Appendix Questionnaire sent to the gynecologist. (DOC)
