# Supplementary material for: Structured reports of pelvic magnetic resonance imaging in primary endometrial cancer: Potential benefits for clinical decision-making
Source: PLoS One. 2019 Mar 25;14(3):e0213928. doi: 10.1371/journal.pone.0213928 (PMC6433257; doi:10.1371/journal.pone.0213928)
Supplement: S1 Appendix — (DOC) [file pone.0213928.s001.doc]

**Appendix 1. Questionnaire sent to the gynecologist**

Please circle answer where appropriate:

Whether the key questions of the referring physician have been answered (on a scale of 1-3)?

1 2 3

Note: 1, yes; 2, results are ambiguous and needing further consultation with radiologists; 3, no.

Whether the information extraction was convenience for you (on a scale of 1-3)?

1 2 3

Note: 1, yes; 2, uncertain or neutral; 3, no.

If you had enough information to make an adequate clinical decision (on a scale of 1-3)?

1 2 3

Note: 1, yes; 2, further consultation with radiologists; 3, no.

How satisfied are you with the linguistic quality of this report (on a scale of 1-5)?

1 2 3 4 5

Note: 1, dissatisfied, misspelling or grammatical mistakes, completely unable to understand; 2, slightly dissatisfied, the description has no obvious error; 3, uncertain or neutral, the description is acceptable; 4, satisfied, the description is clear, pointing out the cancer staging; 5, very satisfied, the description is very clear, pointing out the cancer staging very explicitly.

How satisfied are you with the overall quality of this report (on a scale of 1-5)?

1 2 3 4 5

Note: 1, dissatisfied, the structure is confusion, unable to find information related to the disease; 2, slightly dissatisfied, the structure is slightly confusion, slightly information related to the disease; 3, uncertain or neutral, the structure and content is acceptable; 4, satisfied, the structure is clear, indicating the specific radiological signs associated with patients’ symptoms; 5, very satisfied, the structure is very clear, pointing out the specific radiological signs and diagnosis associated with patients’ symptoms very explicitly.

How much time was needed to read and understand the reports?

___________ (Unit: second)
